# Effectiveness of FitterLife: A Community-Based Virtual Weight Management Programme for Overweight Adults

**DOI:** 10.3390/nu18010017

**Published:** 2025-12-19

**Authors:** Lixia Ge, Fong Seng Lim, Shawn Lin, Joseph Antonio De Castro Molina, Michelle Jessica Pereira, A. Manohari, Donna Tan, Elaine Tan

**Affiliations:** 1 Health Services and Outcomes Research, National Healthcare Group, NHG Health (Annex), 1 Mandalay Road, Singapore 308205, Singapore; joseph.antonio.molina@nhghealth.com.sg (J.A.D.C.M.); michelle.jessica.pereira@nhghealth.com.sg (M.J.P.);; 2 National Healthcare Group Polyclinics, National Healthcare Group, NHG Health (Annex), 1 Mandalay Road, Singapore 308205, Singapore; 3 Clinical Transformation, National Healthcare Group, NHG Health (Annex), 1 Mandalay Road, Singapore 308205, Singaporeelaine.tan@nhghealth.com.sg (E.T.)

**Keywords:** behavioural intervention, body mass index, digital health, primary prevention, programme evaluation, weight management

## Abstract

**Background**: The high prevalence of overweight and obesity in Singapore necessitates scalable primary prevention strategies. This study evaluated the short-term effectiveness of FitterLife, a 12-week, digitally delivered, group-based behavioural weight management programme targeting at-risk adults without diabetes or hypertension in the community. **Methods**: In a retrospective matched cohort study, we compared 306 FitterLife participants (enrolled from October 2021 to January 2025) with 5087 controls identified from a population health data mart, matched on age, sex, ethnicity, and baseline body mass index (BMI). The primary outcome was achieving ≥5% weight loss or a ≥1 kg/m^2^ BMI reduction at 12 weeks. Programme effectiveness was analysed using propensity score matching (1:1) and inverse probability weighted regression. Mixed-effects models assessed weight/BMI trajectories and modified Poisson regression identified behavioural factors associated with success. **Results**: After matching, FitterLife participants were more likely to achieve the weight loss target than controls (45.7% vs. 13.7%, coefficient = 0.32, 95% confidence interval [CI]: 0.26–0.38) and were over three times as likely to succeed (Adjusted incidence rate ratio [aIRR] = 3.37, 95% CI: 2.87–3.93). The programme group showed significant reductions in weight (−2.23 kg, 95% CI: −2.57 to −1.90) and BMI (−0.86 kg/m^2^, 95% CI: −0.95 to −0.73) at the end of programme. Higher session attendance and improved behavioural factors were associated with success. **Conclusions**: FitterLife was effective in achieving clinically significant short-term weight loss in a real-world setting. The findings demonstrate the potential of a scalable, behavioural theory-informed, virtual group model as a viable primary prevention strategy within national chronic disease management efforts.

## 1. Introduction

Overweight and obesity have emerged as a pressing global public health challenge, strongly associated with over 50 medical conditions including type 2 diabetes, hypertension, cardiovascular disease, several cancers, and musculoskeletal disorders [1,2,3]. Beyond health impact, obesity imposes a staggering societal burden: elevated BMI accounted for 3.7 million deaths from non-communicable diseases in 2021 [4], can reduce life expectancy by 5 to 20 years [5,6], and generates substantial economic costs through healthcare expenditure and reduced productivity [3]. Global economic losses linked to overweight and obesity are projected to exceed USD3 trillion annually by 2030 and USD18 trillion by 2060 [4].

The prevalence of overweight and obesity has exhibited a relentless upward trajectory worldwide, driven by problems of modern living factors such as sedentary behaviour, poor dietary practices, and chronic stress. According to the World Health Organization, an estimated 2.5 billion adults (43% of those aged 18 years and older) were overweight (defined as a body mass index (BMI) ≥ 25 kg/m^2^) in 2022, including over 890 million (16%) living with obesity (BMI ≥ 30 kg/m^2^) [4]. This represents a dramatic increase from 1990, when only 25% of adults were overweight [3,7]. Projections indicate that by 2035, 1.9 billion adults (25% of the world’s population) will be living with overweight or obesity, rising further to 3.80 billion (over 50%) by 2050 [7]. Singapore exemplifies this global pattern: the 2022 National Population Health Survey reported a combined overweight and obesity prevalence of 40.2%, of whom 11.6% classified as obese [8]. Alarmingly, despite widespread recognition of this crisis, no country has yet successfully reversed these upward trends [9].

These trends underscore the urgency of developing scalable preventive interventions targeting overweight adults before adverse health conditions such as metabolic diseases develop. Despite evidence that modest weight loss of 5–10% can significantly improve blood pressure, glycaemic control, and lipid profiles [10,11], effective prevention and management remain elusive. Lifestyle modification, comprising dietary changes, increased physical activity, and behavioural support, remains the cornerstone of obesity management. Numerous systematic reviews demonstrate the effectiveness of multi-component behavioural interventions, particularly those incorporating structured coaching and group-based support [12,13,14,15,16,17].

More recently, digital and remote delivery models have gained traction as a viable strategy to enhance the scalability and accessibility of behavioural interventions. Meta-analyses show that eHealth interventions can achieve short-term weight loss comparable to face-to-face programmes [18,19,20]. The remote delivery format can directly address key barriers to participation such as time, travel, and stigma, while still incorporating core behavioural change techniques like structured education, goal-setting, and peer support.

Despite encouraging evidence, weight management programmes continue to face challenges such as high attrition, modest weight loss, and difficulties with long-term adherence. Many are resource-intensive, delivered primarily in healthcare settings, and target individuals with established obesity-related conditions. Although digital and remote behavioural interventions have been associated with improved adherence [20], evidence on the effectiveness of theory-informed, digitally delivered behavioural interventions among overweight adults without diabetes or hypertension, particularly as a scalable primary prevention strategy in Asian community settings, remains limited.

To address this gap, the National Healthcare Group (NHG), one of the three public healthcare clusters in Singapore, launched FitterLife in 2021. Building on a proof-of-concept phase, FitterLife Phase 2 was designed as a 12-week, community-based, virtually delivered weight management programme. It targets overweight adults without diabetes or hypertension and is specifically designed to enable both the activation of key health behaviors and their habituation into sustained daily practice. The programme was explicitly designed for scalability, employing a group-based virtual format to overcome traditional barriers to access. It incorporated key behavioural change techniques including eight weekly interactive sessions via Zoom, covering moderate-to-vigorous physical activity (MVPA), nutrition management, health educational lectures, and group-based goal-setting discussions facilitated by trained health coaches to build skills and self-efficacy. This was followed by three weeks of self-practice with health coaches’ support. Week 12 (session 9) was to address any lapses. Each session lasted two hours. Participants also received guidance on dietary modification (e.g., portion control, fibre intake), physical activity, and behaviour change strategies such as goal-setting and self-monitoring via a secure WhatsApp group throughout the programme period. This is to foster a supportive community and enable continuous self-monitoring and feedback. The overall aims of the FitterLife were to (1) lower the level of obesity by reducing weight, Body Mass Index (BMI) and waist circumference; (2) promote positive lifestyle change through reduction in fat and sugar intake, increase in fibre, wholegrain and vegetable intake, and increase in physical activity level; and (3) increase knowledge and self-efficacy on weight management among at-risk individuals.

This study evaluated the short-term effectiveness of FitterLife phase 2. Specifically, we assessed the likelihood of achieving ≥5% weight loss or ≥1 BMI unit reduction at 12 weeks compared with matched controls, examined weight and BMI trajectories, and investigated the behavioural factors (e.g., attendance, dietary change, physical activity) associated with successful weight loss.

## 2. Methods

### 2.1. Study Design and Data Sources

We conducted a retrospective matched cohort study using data from two sources: (1) programme data systematically collected during FitterLife phase 2 implementation, and (2) retrospectively collected electronic health records from the NHG Population Health Data Mart (PHDM). The PHDM is a comprehensive administrative database that consolidates de-identified individual-level medical, lifestyle, and social data from electronic medical records, laboratory results, clinical registries, and health surveys across NHG’s network of healthcare and social care providers and partners.

This study was conceived after the conclusion of the FitterLife programme; therefore, neither the programme nor this study was prospectively registered. To ensure transparency and reduce the risk of analytical bias, the key methodological components, including the primary and secondary outcomes, eligibility criteria, matching strategy, and statistical approach, were explicitly defined prior to data extraction and analysis, based on the programme’s predefined goals.

### 2.2. Study Participants

**FitterLife (Intervention) Cohort:** The exposed group consisted of 360 community-dwelling individuals enrolled in FitterLife phase 2 between October 2021 and January 2025. Eligibility criteria were: (1) Singapore residents aged 21–64 years, (2) overweight (BMI ≥ 23.0 kg/m^2^, based on Asian cut-offs), (3) no prior diagnosis of diabetes or hypertension, and (4) not pregnant and free from active medical conditions that could impede exercise, including cardiac, respiratory, musculoskeletal, or psychiatric issues, for the past six months. Recruitment prioritised ethnic minority and lower socioeconomic status subgroups to ensure programme accessibility and representation. The programme was delivered over 20 runs, with cohort sizes ranging from 8 to 30 participants per run. After excluding ineligible recruits (n = 14), withdrawals (n = 15), and those with incomplete baseline (n = 3) or follow-up data (n = 22), the final intervention cohort for analysis was 306 individuals.

**Figure 1:**
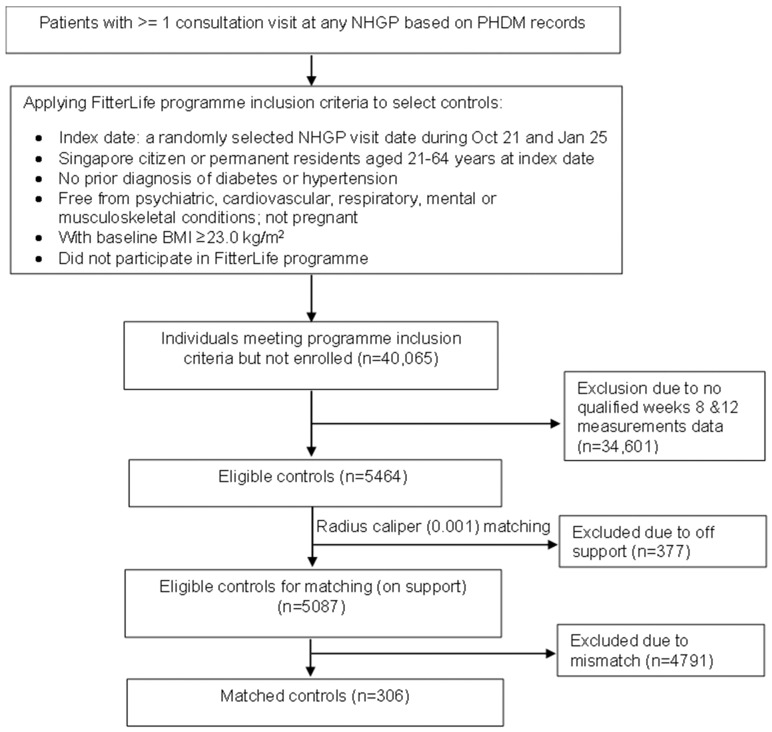
Control cohort selection flowchart.

**Control Cohort:** The unexposed controls were selected from the PHDM database using the same inclusion and exclusion criteria during the identical study period. To be eligible, controls must have had at least one visit to any NHG Polyclinic (NHGP), a group of polyclinics providing subsidised primary care in Singapore, but no record of enrolment in FitterLife. A random visit date was designated as the index enrolment date for each control to establish a temporal reference point analogous to the enrolment date for FitterLife participants. The initial control pool meeting these criteria comprised 5464 individuals (Figure 1).

### 2.3. Sample Size Considerations

As a retrospective study using existing programme and administrative data, the sample size was determined by the number of eligible FitterLife participants (n = 306). To assess whether this sample provided adequate statistical power, a post hoc power analysis [21] was conducted for the primary binary outcome (≥5% weight loss or ≥1 kg/m^2^ BMI reduction). Based on the observed success rates of 45.8% in the FitterLife cohort and 13.6% in the matched control cohort, with a two-sided alpha of 0.05, the study achieved >99.9% power to detect this clinically meaningful difference. This confirms that the study was adequate to detect the difference observed.

### 2.4. Outcome Measures

The primary outcome was achievement of meaningful weight loss at week 12, defined as either a ≥5% reduction in body weight or a ≥1 kg/m^2^ reduction in BMI. This composite outcome balances international evidence with population relevance: the ≥5% weight loss threshold is the established benchmark for clinically meaningful cardiometabolic improvement [22], while the ≥1 kg/m^2^ BMI reduction criterion captures significant, achievable weight loss as a pragmatic public health target for lifestyle interventions, particularly relevant in populations with elevated metabolic risk at lower BMI [23]. Secondary outcomes included absolute weight and BMI at weeks 1, 4, 8, 12, and 36 as well as changes from baseline in these measures at weeks 4, 8, 12, and 36.

Data for FitterLife participants were obtained retrospectively from anonymised programme records containing self-reported weight and height measurements collected upon recruitment and during routine programme monitoring. To ensure internal consistency for capturing change over time, participants were instructed to use the same weighing scale throughout the programme. They submitted photographs of their weight scale readings to a secured WhatsApp line accessible only to two programme coordinators via a designated, password-protected mobile phone for subsequent verification and documentation.

To assess the reliability of these self-reported measurements, self-reported weight and height values were cross-checked against available measurements in the PHDM recorded for the same participant within a comparable period. As discrepancies were minimal (≤1 kg) and the self-reported data provided a more complete time-series aligned with programme data points, the self-reported measurements were retained as the primary data source.

For participants missing follow-up weight data at the predefined study timepoints, these values were supplemented, where available, with corresponding PHDM measurements. This approach was justified by the minimal observed discrepancies in baseline weights between the two sources. For controls, all weight and BMI data were obtained directly from the PHDM, with baseline values defined as measurements taken within 120 days before or up to 14 days after enrolment, and follow-up values defined as those recorded within ±14 days of each target week.

Percentage weight change at week 12 was calculated as follows: [(weight at week 12 − baseline weight)/baseline weight] × 100. The absolute change in BMI was calculated as BMI at week 12 − baseline BMI. For participants in either cohort missing week 12 data, week 8 measurements were carried forward (last observation carried forward).

### 2.5. Demographic and Behavioural Variables

Demographic factors including age, sex, and ethnicity were obtained for both cohorts from PHDM. For the FitterLife cohort, self-reported behavioural data at weeks 1 and 12 including time spent on MVPA per week and walking per day (assessed using questions adapted from the International Physical Activity Questionnaire [24]), and dietary patterns (measured by fat and fibre intake collected using questions adapted from the fat and fibre behaviour questionnaire [25]) were retrieved from anonymised FitterLife programme records, which were collected via FormSG (a secure government-supported platform used for encrypted data collection in Singapore). These behavioural data were used exclusively to explore factors associated with successful weight loss within the FitterLife group and were not included in the comparative effectiveness analysis against controls.

Data on programme engagement (e.g., session attendance) and operating costs were also obtained (reported in Supplementary File S1).

### 2.6. Statistical Analysis

#### 2.6.1. Descriptive Statistics and Exploratory Analysis

Baseline characteristics were summarised using frequencies (n) and percentages (%) for categorical variables and means with standard deviations (SD) for continuous variables. Group comparisons before and after matching were conducted using Chi-square tests for categorical variables and independent *t*-tests for continuous variables. The absolute proportion of individuals who achieved the weight loss target was calculated for both groups after matching. Additionally, programme cost per kilogram of weight loss was calculated as a descriptive indicator of programme efficiency (reported in Supplementary File S1).

#### 2.6.2. Propensity Score Matching (PSM)

To account for baseline confounding, propensity scores were estimated via logistic regression using age, sex, ethnicity, and baseline BMI as predictors of programme enrolment. Analyses were restricted to the region of common support [26], excluding 377 controls outside this range (Figure 1). Participants were matched 1:1 using nearest-neighbour matching without replacement and a caliper width of 0.001. This narrow caliper was selected to ensure close matches and minimise residual bias, made feasible by the large pool of potential controls. Covariate balance was assessed using standardised mean differences (<0.10 indicating good balance) and variance ratios (0.5–2.0 indicating adequate balance).

#### 2.6.3. Primary Effectiveness Analysis

The average treatment effect on the treated (ATET) for achieving the weight loss target was estimated using inverse probability weighted regression adjustment (IPWRA) with robust standard errors. This doubly robust estimator combines propensity score weighting with outcome regression to provide unbiased effect estimates under either correct specification. Results were expressed as risk differences (coefficients) with 95% confidence intervals (CIs). Sensitivity analysis using modified Poisson regression were performed on both unmatched and matched cohorts to estimate relative risks (adjusted incidence rate ratios, IRRs) with 95% CIs, adjusting for baseline covariates (age, female, Chinese, and baseline BMI).

#### 2.6.4. Longitudinal Trajectory Analyses

To assess changes in weight and BMI over time, mixed-effects linear regression models were applied to the trimmed control cohort (before matching), analysing both absolute values and changes from baseline at each follow-up. Models included fixed effects for treatment group, baseline covariates (age, sex, ethnicity, baseline BMI), categorical time points (weeks 1, 4, 8, 12, and 36), and group-by-time interaction terms, with random intercepts for individuals. An unstructured variance-covariance matrix was specified to account for within-person correlations.

#### 2.6.5. Predictors of Success Analysis

Within the FitterLife cohort, modified Poisson regression was used to identify behavioural factors associated with successful weight loss (≥5% body weight or ≥1 kg/m^2^ BMI reduction) at week 12. Predictors included session attendance, and change in physical activity and dietary intake, adjusting for age, sex, ethnicity, and baseline BMI. Multicollinearity was assessed using variance inflation factors (VIF < 5.0 indicating no substantive concern) [27].

All analyses were conducted using Stata SE 18.0, with statistical significance set at *p* < 0.05.

### 2.7. Ethical Considerations

This study was determined to be exempt from full ethics review (“review not required”) as it involved the analysis of existing, anonymised programme and administrative data, posed on no more than minimal risk, the original consent specifically covered this use, and re-identification or re-contacting participants was impracticable. Formal approval for the study conduct was granted by the NHG Group Research & Innovation Office on 8 May 2025. All data handling complied with the Singapore Personal Data Protection Act (PDPA) and NHG’s institutional data governance policies.

TheFitterLife programme is a free-of-charge, evidence-based weight management service delivered by a community care team from NHG. Access to identifiable information was strictly restricted to two programme coordinators and the health coach to facilitate communication and personalised progress monitoring. Prior to registration, all participants provided informed consent for data collection and the future use of their anonymised data for evaluation and research.

For this study, the linkage of participant records (via National Registration Identity Card number) to the PHDM was performed by a designated trusted third party to preserve confidentiality. Following this linkage, all data were fully anonymised for analysis. The final analysis dataset was stored on a secure, password-protected corporate laptop, accessible only to the study data analyst, who had no access to identifiable information.

## 3. Results

### 3.1. Participant Characteristics and Propensity Score Matching

Before matching, the mean age of the 306 FitterLife participants was 47.8 (SD: 10.7) years, which was comparable to the control group (*p* = 0.676). The majority were females (77.8%) and Chinese (83.3%), both proportions higher than in the control group (both *p* < 0.001). Baseline weight was 73.6 (SD: 12.1) kg and BMI was 28.1 (SD: 3.6) kg/m^2^, with BMI significantly higher than the control group mean of 27.2 kg/m^2^ (*p* < 0.001).

After matching, all variables achieved adequate balance with non-significant differences (Table 1). The absolute standardised mean difference was less than 5% for all variables, ranging from −2.0% for age to 4.2% for baseline BMI. Variance ratios for baseline age (0.84) and BMI (1.05) within the recommended range of 0.5–2.0, confirming successful matching.

### 3.2. Effectiveness on Weight Loss Target

The FitterLife programme significantly increased the probability of achieving the weight loss target by 32 percentage points (ATET = 0.32, 95% CI: 0.26, 0.38, *p* < 0.001). Specifically, 45.7% of participants achieved success, compared to a counterfactual estimate of 13.7% in the absence of the programme.

These findings were consistent across analytical approaches. Modified Poisson regression demonstrated that participants in the FitterLife programme were 3.32 times more likely to achieve weight loss target at week 12 in the unmatched cohort (aIRR = 3.32, 95% CI = 2.85, 3.86, *p* < 0.001), with similar results in the matched cohort (aIRR = 3.37, 95% CI = 2.87, 3.93, *p* < 0.001) (Table 2).

### 3.3. Trajectories of Weight and BMI Change

**Figure 2:**
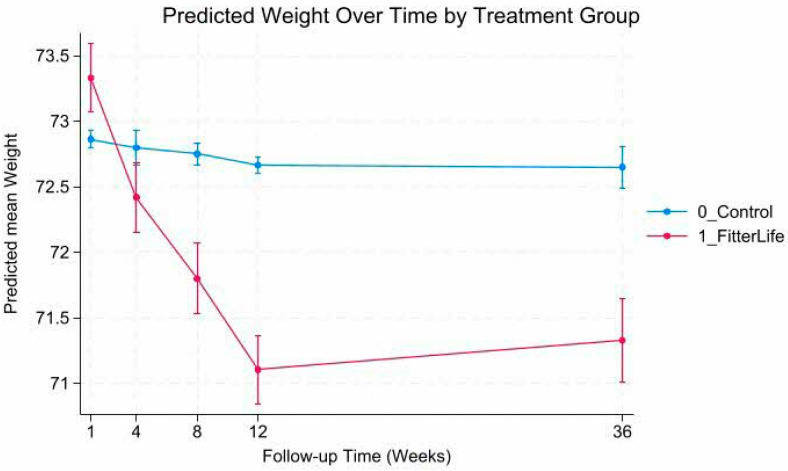
Predicted weight trajectories by treatment group.

**Figure 3:**
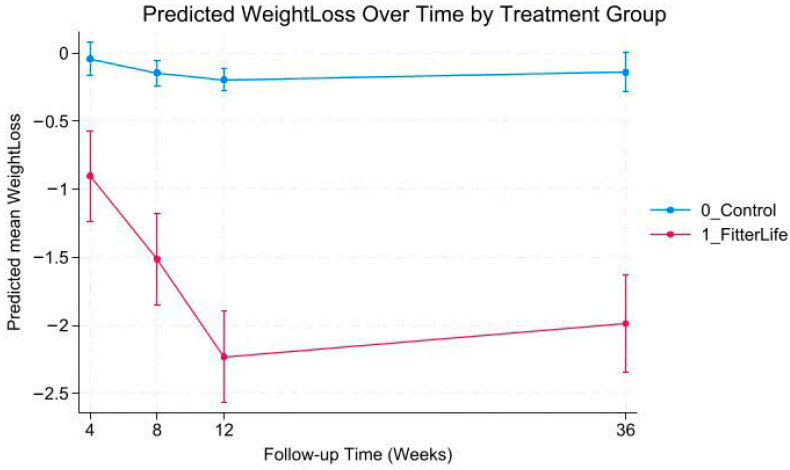
Predicted weight change trajectories by treatment group.

**Figure 4:**
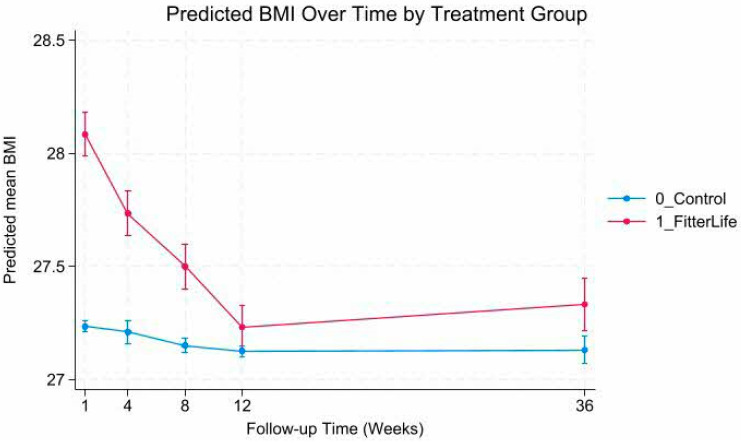
Predicted BMI trajectories by treatment group.

**Figure 5:**
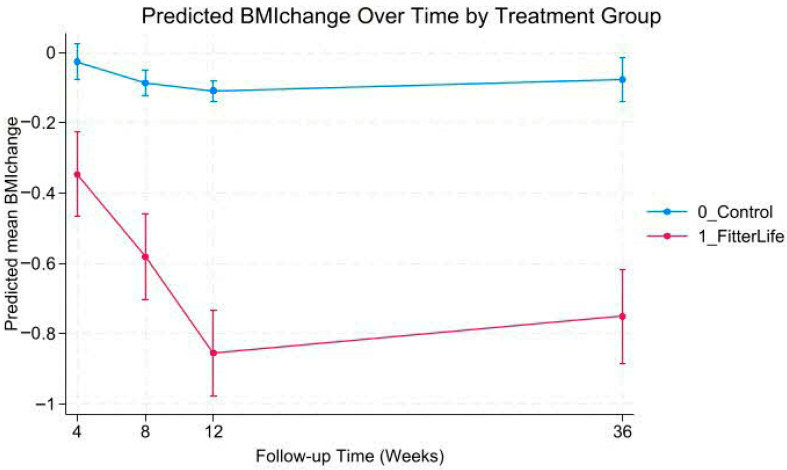
Predicted BMI change trajectories by treatment group.

Mixed-effects models revealed a statistically significant group-by-time interaction (*p* < 0.001), indicating significant differences in weight and BMI trajectories between FitterLife and Controls over 36 weeks (Figure 2, Figure 3, Figure 4 and Figure 5). The FitterLife group demonstrated progressive weight and BMI reductions throughout the intervention period, with maximum reductions observed at week 12 (end of programme): with a mean weight loss of −2.23 kg (95% CI: −2.57, −1.90) and a BMI reduction of −0.86 kg/m^2^ (95% CI: −0.95, −0.73). In contrast, controls showed minimal change (−0.19 kg and −0.11 kg/m^2^, respectively). Although some weight regains occurred post intervention, the FitterLife group maintained meaningful reductions at week 36 (weight change: −1.99 kg [95% CI: −2.34, −1.63], BMI change: −0.75 kg/m^2^ [95% CI: −0.89, −0.62]).

### 3.4. Behavioural Factors Associated with Weight Loss Success

Higher session attendance (7–9 sessions), dietary improvements (≥20% fat reduction, increased fibre intake), and increased physical activity (≥1 h MVPA weekly) were associated with greater likelihood of achieving week 12 weight loss target among FitterLife participants (Table 3).

## 4. Discussion

This retrospective matched cohort study evaluated the real-world effectiveness of FitterLife, a scalable, digitally delivered, community-based behavioural intervention for overweight adults in Singapore without diabetes or hypertension. Our results showed that programme participation was associated with a significantly higher likelihood of achieving successful weight loss (≥5% weight or ≥1 kg/m^2^ BMI reduction) at week 12 compared to matched controls, with an adjusted incidence rate ratio of 3.37, representing 32-percentage point increase in success probability. Furthermore, mixed-effects models confirmed a superior trajectory of weight and BMI reduction throughout the intervention period. Critically, our within-group analysis provides insights into the behavioural mechanisms of change, demonstrating that higher programme engagement and positive behavioural changes were key drivers of success.

The FitterLife programme demonstrated substantial effectiveness, with 45.7% participants achieving successful weight loss, a success rate comparable to other real-world lifestyle interventions [12,13,28]. The mean absolute weight loss of 2.23 kg at week 12 aligns with established efficacy of short-term, multi-component interventions (a pooled mean change of −2.70 kg) [16]. These findings collectively support the evidence that short-term, multi-component lifestyle interventions may be associated with modest but clinically meaningful weight loss [29,30].

Our findings add to the growing body of evidence supporting digital and remote delivery models for behavioural interventions [18,19,20]. The programme’s virtual, group-based format likely enhanced accessibility and scalability while actively leveraging behavioural principles. The use of live, interactive Zoom sessions and WhatsApp groups facilitated a sense of community and provided timely support, key elements for maintaining engagement and self-efficacy in remote interventions. This model is particularly suited for a busy, multi-ethnic urban populations like Singapore’s, where barriers such as time and travel can hinder participation in traditional face-to-face programmes [19,31]. Our study provides robust evidence that such a digitally enabled, theory-informed approach is effective in an Asian, community-based preventive population—an underrepresented group in prior research.

Our analysis of the FitterLife cohort offers valuable insights into behavioural mechanisms underpinning its effectiveness. The dose–response relationship between session attendance (7–9 sessions) and successful weight loss underscores that engagement with the behavioural content and group process is a critical active ingredient and highlights the critical importance of programme engagement and adherence, a consistent finding across behavioural science [32,33]. Furthermore, the independent association between increased fibre intake and weight loss success highlights the pivotal role of targeting specific, actionable dietary behaviours beyond mere caloric restriction [34,35,36]. Collectively, these findings reinforce the value of the programme’s multi-component behavioural approach, which simultaneously targeted knowledge, skills, self-monitoring, and social support to facilitate change across multiple lifestyle domains.

### 4.1. Strengths and Limitations

The primary strength of this study lies in its retrospective comparative effectiveness design, which leverages a large, well-characterised control pool. The application of propensity score matching ensured a high degree of comparability between groups on observed baseline characteristics, substantially reducing measured confounding and strengthening causal inference. Furthermore, the use of mixed-effects models for longitudinal analysis accounted for within-individual correlations and provided a nuanced assessment of weight trajectories over time.

However, several limitations warrant consideration. First, despite rigorous matching, the observational, non-randomised design cannot preclude residual confounding by unmeasured factors, such as underlying motivation, socioeconomic status, or social support, which may influence both programme enrolment and weight loss success. Second, the primary reliance on self-reported weights (via personal scales) for the FitterLife cohort, despite validation efforts, introduces a potential for measurement bias not present in the clinically sourced control data. Third, detailed behavioural data (diet and physical activity) were self-reported for the FitterLife cohort and are therefore subject to social desirability bias. Furthermore, lack of such data for the control cohort limits the analysis of these behavioural factors as potential mediators or confounders of the observed outcomes. Fourth, the 36-week follow-up period is insufficient to assess long-term weight maintenance; the observed trend of weight regain at week 36 underscores the challenge of sustainability and highlights the need for future research to evaluate the durability of effects and the potential role of structured maintenance programmes.

### 4.2. Programme Implications and Future Directions

Our findings support the potential of the FitterLife as a scalable, digitally delivered intervention for primary weight management in community settings. To translate this promise into sustained public health impact, future implementation must address two key challenges derived from our evaluation:

1. Enhancing Programme Sustainability: The trend of weight regain by 36 weeks indicates a critical need to integrate structured maintenance phases or booster sessions, informed by behavioural theory, to improve long-term outcomes.
2. Optimising Implementation and Reach: The programme’s broader impact is currently constrained by low recruitment, which resulted in a high operating cost ($988.04 in Singapore dollars per participant; SGD $443.07 per kilogram lost). Addressing this requires deeper integration with primary care networks and community partnerships to improve cost-efficiency and scale.

For future research, three key priorities emerge to build on these findings: (1) a full cost-effectiveness analysis from a public health perspective; (2) longer-term follow-up to assess the durability of weight loss and its impact on hard endpoints like the incidence of diabetes and hypertension; and (3) mixed-methods research to elucidate the behavioural mechanisms of engagement and dropout, providing a direct evidence base for refining the intervention’s behavioural components and digital delivery.

## 5. Conclusions

In conclusion, this evaluation provides evidence supporting the short-term effectiveness of the FitterLife programme in a real-world community setting. The virtual group-based delivery model appears viable, and the outcomes observed were associated with adherence and improvements in behavioural factors. While challenges in recruitment and cost-efficiency persist, these findings support the case for continued investment and systematic optimisation of such scalable interventions.

## Figures and Tables

**Table 1:** Baseline characteristics before and after matching.

Baseline Characteristics	Before Matching		*p*-Value	After Matching		*p*-Value
	FitterLife	Control		FitterLife	Control	
	n = 306	n = 5087		n = 306	n = 306	
**Age in years, mean ± SD**	47.8 ± 10.7	47.5 ± 11.9	0.676	47.8 ± 10.7	48.1 ± 11.6	0.805
**Gender, n (%)**			<0.001			0.876
Male	68 (22.2)	2265 (44.5)		68 (22.2)	66 (21.6)	
Female	238 (77.8)	2822 (55.5)		238 (77.8)	240 (78.4)	
**Chinese ethnicity, n (%)**	255 (83.3)	3563 (70.0)	<0.001	255 (83.3)	257 (83.9)	0.853
**Weight in kg, mean ± SD**	73.6 ± 12.1	72.9 ± 12.2	0.325	73.6 ± 12.1	72.7 ± 12.0	0.125
**BMI in kg/m^2^, mean ± SD**	28.1 ± 3.6	27.2 ± 3.2	<0.001	28.1 ± 3.6	28.0 ± 3.5	0.605

Abbreviations: BMI—body mass index; SD—standard deviation.

**Table 2:** Effectiveness of FitterLife on Weight Loss at Week 12.

Statistical Methods	Outcome: Achieved Weight Loss Target at Week 12 (≥5% Reduction in Weight or ≥1 kg/m^2^ Reduction in BMI)				
	Control	FitterLife	*p*-Value	Coefficient/Adjusted Incidence Rate Ratio (Ref: Control)	95% Confidence Interval
IPWRA (ATET)	13.7%	45.7%	<0.001	0.32	0.26, 0.38
Modified Poisson regression on unmatched sample *	644 (12.7%)	140 (45.8%)	<0.001	3.32	2.85, 3.86
Modified Poisson regression on matched sample *	42 (13.6%)	140 (45.8%)	<0.001	3.37	2.87, 3.93

* Adjusted for age, female, Chinese, and baseline BMI. Abbreviations: ATET—average treatment effect on the treated; BMI—body mass index; IPWRA—inverse probability weighted regression adjustment.

**Table 3:** Behavioural factors associated with successful weight loss at week 12 (n = 290).

Behavioural Factor	Outcome: Achieved Weight Loss Target at Week 12 (≥5% Reduction in Weight or ≥1 kg/m^2^ Reduction in BMI)			
	n (%)	*p*-Value	Adjusted IRR (95% CI)	*p*-Value
**Sessions attended**		0.001		0.011
2 to 6 sessions (n = 68)	20 (29.4%)		Ref.	
7 to 9 sessions (n = 222)	115 (51.8%)		1.63 (1.12, 2.37)	
**Change in fat intake score**		0.057		
No change or increase (n = 61)	21 (34.4%)		Ref.	
0 to <20% decrease (n = 166)	79 (47.6%)		1.46 (0.99, 2.16)	0.057
≥20% decrease (n = 63)	35 (55.6%)		1.66 (1.14, 2.42)	0.041
**Change in fibre intake score**		0.001		
No change or decrease (n = 68)	20 (29.4%)		Ref.	
Increase (n = 222)	115 (51.8%)		2.58 (1.30, 5.14)	0.007
**Change in weekly MVPA**		0.013		
No change or decrease (n = 136)	56 (41.2%)		Ref.	
0 to <1 h increase (n = 69)	28 (41.6%)		1.26 (0.88, 1.81)	0.202
≥1 h increase (n = 85)	51 (60.0%)		1.66 (1.24, 2.23)	0.001
**Change in daily walking time**		0.079		
No change or decrease (n = 118)	48 (40.7%)		Ref.	
0 to <16 min increase (n = 79)	35 (44.3%)		1.03 (0.73, 1.47)	0.859
≥16 min increase (n = 93)	52 (55.9%)		1.77 (0.95, 1.70)	0.104

Adjusted for age, sex, ethnicity, and baseline BMI. Abbreviations: BMI—body mass index; 95% CI: 95% confidence interval; IRR—incidence rate ratio; MVPA—moderate-to-vigorous physical activity; Ref.—reference.

## Data Availability

The data supporting this study’s findings are not publicly available due to sensitivity concerns but may be obtained from the corresponding author upon reasonable request.

## Supplementary Materials

The following supporting information can be downloaded at: https://www.mdpi.com/article/10.3390/nu18010017/s1, File S1: Programme Engagement and Operating Costs.

## Abbreviations

aIRR	Adjusted incidence rate ratio
ATET	Average treatment effect on the treated
BMI	Body mass index
95% CI	95% confidence interval
IPWRA	Inverse probability weighted regression adjustment
MVPA	Moderate-to-vigorous physical activity
NHG	National Healthcare Group
NHGP	National Healthcare Group Polyclinic
PHDM	NHG Population Health Data Mart
PSM	Propensity score matching
Ref.	Reference
SD	Standard deviation
VIFs	Variance inflation factors
