# Supplementary material for: Effectiveness of FitterLife: A Community-Based Virtual Weight Management Programme for Overweight Adults
Source: Nutrients. 2025 Dec 19;18(1):17. doi: 10.3390/nu18010017 (PMC12787922; doi:10.3390/nu18010017)
Supplement: Supplementary file 1 [file nutrients-18-00017-s001.zip › nutrients-4002683-supplementary.pdf]

File S1.

Programme Engagement and Operating Costs

The programme demonstrated high retention, with a low dropout rate of 4.2% (15/360). The average session attendance rate was 75.5% (range: 71.2% to 83.4%), corresponding to a mean of 6.8 out of 9 sessions attended for all enrolled participants. Attendance was higher in the final analysis cohort (n=306), with a mean of 7.4 sessions.

The estimated mean programme operating cost was S$988.04 per enrolled participant. Based on the mean weight loss of 2.23 kg at 12 weeks, the resulting operating cost per kilogram of weight loss per participant was S$443.07.
